# Supplementary material for: Comparison of anorectal function measured using wearable digital manometry and a high resolution manometry system
Source: PLoS One. 2020 Sep 29;15(9):e0228761. doi: 10.1371/journal.pone.0228761 (PMC7523952; doi:10.1371/journal.pone.0228761)
Supplement: S1 Text — (DOCX) [file pone.0228761.s001.docx]

**S1 Text: Schematics of the portions of the wrist-mounted determining unit hardware that involved the pressure and myoelectric signal processing.**


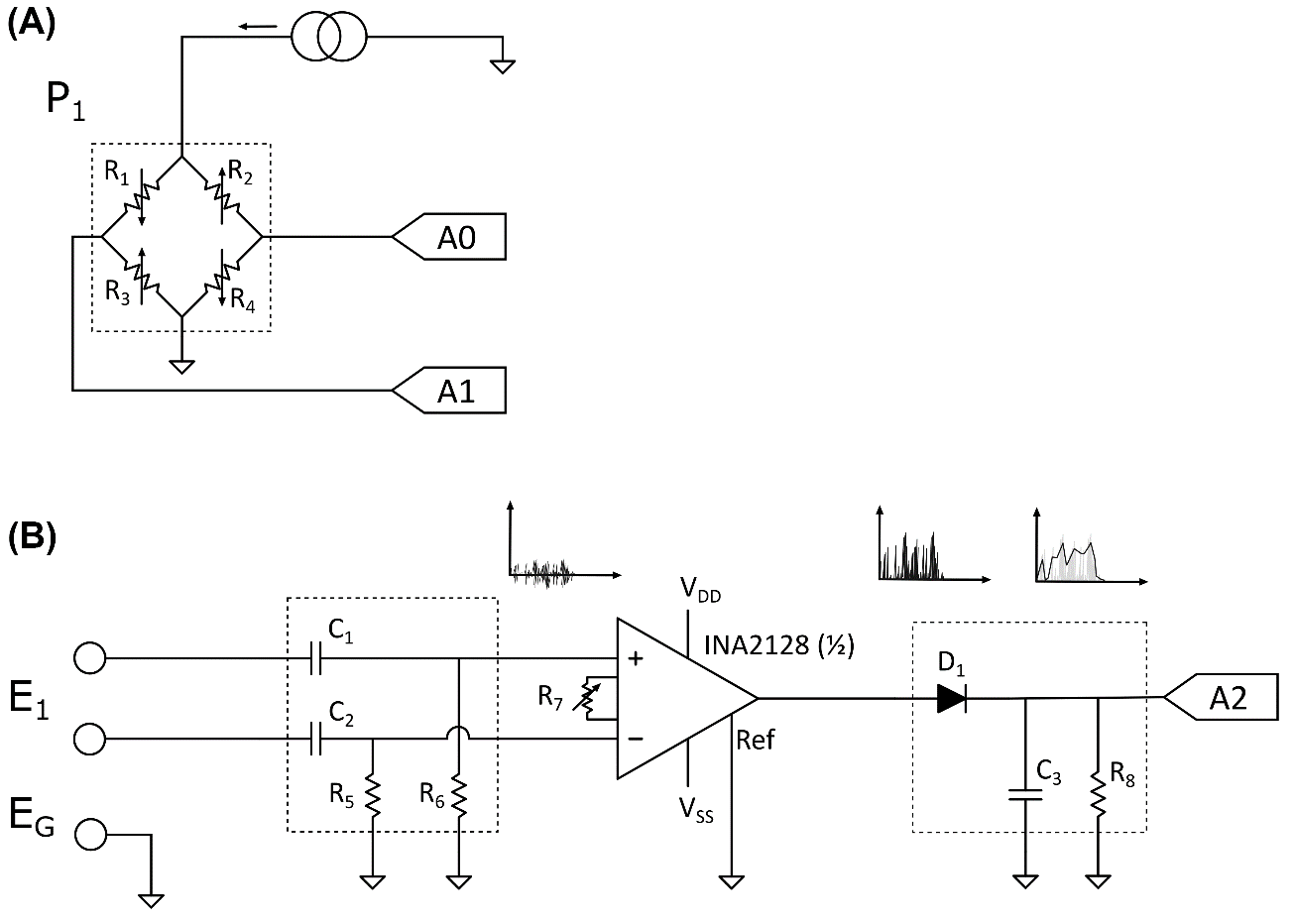


1. Schematic of the pressure sensing circuitry for each of the pressure sensors (i.e., P_1-3_). A 5 mA constant current source powers the piezo-resistive (P1602) pressure sensor. The following values were used for components: R_1_, R_2_, R_3_ and R_4_ = 5 kΩ. A0 and A1 were connected to a 12 bit analog to digital converter (e.g., using [Adafruit nrf52840](https://www.adafruit.com/product/4062)). The difference of A0 and A1 signals changes linearly with the pressure. The difference voltage was large enough to be measured directly using the analog to digital converter.
2. Simple schematic of the myoelectric signal amplifier for the myoelectric electrodes (i.e., E_1-2_). E_G_ represents the body ground electrode. Signals were passed through the first order high pass filter (f_c_ = 186 Hz), amplified ([INA2128](http://www.ti.com/lit/ds/sbos035a/sbos035a.pdf)), rectified (D_1_), and finally modulated using a simple RC envelope detector (C_3_ and R_8_). A2 is another 12 bit analog to digital converter. The following values were used for components: C_1 and 2_ = 22 nF, R_5 and 6_ = 39 kΩ, R_7_ = 20 Ω, C_3_ = 4.7 µF and R_8_ = 10 kΩ. The input pins of the INA2128 are automatically protected from electrostatic discharges via the hardware design.
